# Genomic and Functional Characterization of *Enterococcus faecalis* Isolates Recovered From the International Space Station and Their Potential for Pathogenicity

**DOI:** 10.3389/fmicb.2020.515319

**Published:** 2021-01-11

**Authors:** Noelle C. Bryan, Francois Lebreton, Michael Gilmore, Gary Ruvkun, Maria T. Zuber, Christopher E. Carr

**Affiliations:** ^1^Department of Earth, Atmospheric and Planetary Sciences, Massachusetts Institute of Technology, Cambridge, MA, United States; ^2^Department of Ophthalmology, Massachusetts Eye and Ear Infirmary, Boston, MA, United States; ^3^Walter Reed Army Institute of Research, Silver Spring, MD, United States; ^4^Department of Molecular Biology, Massachusetts General Hospital, Boston, MA, United States; ^5^Georgia Institute of Technology, Atlanta, GA, United States

**Keywords:** International Space Station (ISS), *Enterococcus faecalis*, pangenome, antibiotic resistance, desiccation tolerance, pathogenicity

## Abstract

*Enterococcus faecalis* is a multidrug resistant, opportunistic human pathogen and a leading cause of hospital acquired infections. Recently, isolates have been recovered from the air and surfaces onboard the International Space Station (ISS). Pangenomic and functional analyses were carried out to assess their potential impact on astronaut health. Genomes of each ISS isolate, and both clinical and commensal reference strains, were evaluated for their core and unique gene content, acquired antibiotic resistance genes, phage, plasmid content, and virulence traits. In order to determine their potential survival when outside of the human host, isolates were also challenged with three weeks of desiccation at 30% relative humidity. Finally, pathogenicity of the ISS strains was evaluated in the model organism *Caenorhabditis elegans.* At the culmination of this study, there were no defining signatures that separated known pathogenic strains from the more commensal phenotypes using the currently available resources. As a result, the current reliance on database information alone must be shifted to experimentally evaluated genotypic and phenotypic characteristics of clinically relevant microorganisms.

## Introduction

*Enterococcus faecalis* represent a core, yet quantitatively minor portion of the human gut microbiome (Schloissnig et al., 2013) that are well suited to persist outside of the host environment. Evidence recently emerged that the enterococci may have split from their last common ancestor at approximately the time animal life began to colonize terrestrial habitats (∼425 million years ago), and traits that promote survival and transmission in the exposed land environment selected for the characteristic ruggedness of the genus (Lebreton et al., 2017). Supporting that proposition, enterococci display significantly higher levels of resistance to a variety of antiseptics, salts, organic compounds, desiccation, and starvation than ancestral outgroups (Lebreton et al., 2017).

Because traits contributing to environmental persistence also contribute to persistence in the hospital environment, enterococci rank among leading causes of healthcare associated infections (Weiner et al., 2016). While certain strains of *E. faecalis* are pathogenic for hospitalized patients, *e.g*., MMH594 (Huycke et al., 1991) and V583 (Sahm et al., 1989), and possess genomes swollen to 3.3 Mb by the accretion of mobile genetic elements (MGEs), the genomes of commensal isolates are 25% smaller, as typified by the strain OG1RF (Bourgogne et al., 2008). In the antibiotic era, loss of CRISPR (clustered regularly interspaced short palindromic repeats) protection of the chromosome further facilitated the accumulation of additional antibiotic resistance by enterococci (Fiore et al., 2019). Several other MGEs have been directly linked to virulence factors (Shankar et al., 2002), however, the selective value of most mobile elements in clinical isolates of enterococci remains to be determined.

The ISS provides a unique opportunity to study the establishment of a microbiome in what originated as a clean, hermetically sealed built environment, where the influx of new microorganisms only occurs periodically with the arrival of new crew and supplies. The microbiome of the ISS has been extensively characterized in recent years by metagenomic (Be et al., 2017; Lang et al., 2017; Singh et al., 2018b; Urbaniak et al., 2018; Blaustein et al., 2019; Mora et al., 2019; Sielaff et al., 2019) and culture-based investigations (Schiwon et al., 2013; Venkateswaran, 2017; Singh et al., 2018a; Blaustein et al., 2019; Mora et al., 2019; Sobisch et al., 2019; Urbaniak et al., 2019), yet only recently have these effort been combined (Mora et al., 2019; Sielaff et al., 2019). The results are divided among those that report similarities to Earth built environments (Schiwon et al., 2013; Mora et al., 2019; Sielaff et al., 2019) including United States based spacecraft assembly cleanrooms (Be et al., 2017), and those that detected significant differences from terrestrial residences, the Human Microbiome Project (Lang et al., 2017), the Japan-based ISS analog module (Ichijo et al., 2016), and a French Guiana-based cleanroom (Mora et al., 2019). Despite the differences in methodologies and results, the detection of opportunistic human pathogens (Schiwon et al., 2013; Be et al., 2017; Lang et al., 2017; Mora et al., 2019; Singh et al., 2018a,b; Sielaff et al., 2019; Sobisch et al., 2019; Urbaniak et al., 2019) including *E. faecalis*, is a common phenomenon. With the exception of Be et al. (2017), the previous metagenomic efforts were limited to characterizing diversity at the genus level or identifying antibiotic resistance or virulence gene content, and thereby lacked the genetic resolution to confidently identify pathogenic bacterial strains. While genome-based pathogenicity analyses have been performed for *Staphylococcus aureus* (Blaustein et al., 2019) and *Enterobacter bugandensis* (Singh et al., 2018a), only a single culture-based report has quantified pathogenicity (*Fusarium oxysporum* isolates from the ISS Urbaniak et al., 2019) in a host model. As a result, the ability to predict how opportunistic pathogens may impact crew health remains unclear.

In addition to characterizing the microbiome of the ISS, understanding the effects of spaceflight on bacterial physiology will be imperative for assessing the impacts on crew health. When *Pseudomonas aeruginosa* was exposed to spaceflight, there was an increase in the total cell viable numbers, biomass, biofilm thickness, and the cells produced a unique biofilm architecture not seen in ground controls of the species (Mclean et al., 2001; Kim et al., 2013). Su et al. (2014) documented growth curve, transcriptomic, and proteomic alterations (related to amino acid transport, metabolism, energy production, and conversion) in *Bacillus cereus* and *Serratia marcescens* during spaceflight (Wang et al., 2014). Carbon utilization profiles were altered among *S. marcescens* clones after flight, and transcriptomic and proteomic data revealed significant changes in metabolic functions (Wang et al., 2014). Additionally, Wilson et al. (2007) utilized proteomic analysis and expression profiles to demonstrate that changes in genomic regulation of *Salmonella typhymurium* were widely distributed, and virulence was increased in response to spaceflight. Although Hammond et al. (2013) report a significant decrease in *E. faecalis* OG1RF virulence when grown under spaceflight conditions, this was inferred by a reduction in the optical density of cultures grown in the presence of adult *Caenorhabditis elegans* rather than worm viability.

In addition to altered microbial physiological responses, astronaut health is also impacted by the spaceflight environment. Factors such as microgravity, physiological stress, isolation, abnormal circadian rhythms, and altered nutrition have been shown to weaken an astronaut’s immunity in as little as 10 to 15 days (Crucian et al., 2013), and these effects can persist up to 6 months post mission ISS (Crucian et al., 2015). This weakened immunity, combined with the prevalence of *E. faecalis* strains, or other potential pathogens including viruses (Rooney et al., 2019), could result in severe human health consequences on board the ISS, even in incidences of routine medicinal procedures (*i.e*., the introduction of a percutaneous catheter Mermel, 2012).

With the detection of enterococci, we sought to determine the risk they posed to residents within the ISS – specifically, do they represent pathogenic lineages commonly associated with infections in hospitals, or do they represent commensals shed into the environment as a consequence of human habitation? In order to determine if the challenging environment of the air and surfaces of the ISS have selected for pathogenic strains of *E. faecalis*, we coupled previously employed genomic data analyses (Singh et al., 2018a) with phenotypic characterization of *E. faecalis* isolates recovered onboard the ISS. By assessing their antibiotic resistance, desiccation tolerance, and pathogenicity in the well-established *C. elegans* model (Garsin et al., 2001), we sought to test existing methods for identification of strains of *E. faecalis* with a high likelihood of causing in-flight infections.

## Materials and Methods

### Strains and Culture Conditions

**FIGURE 1:**
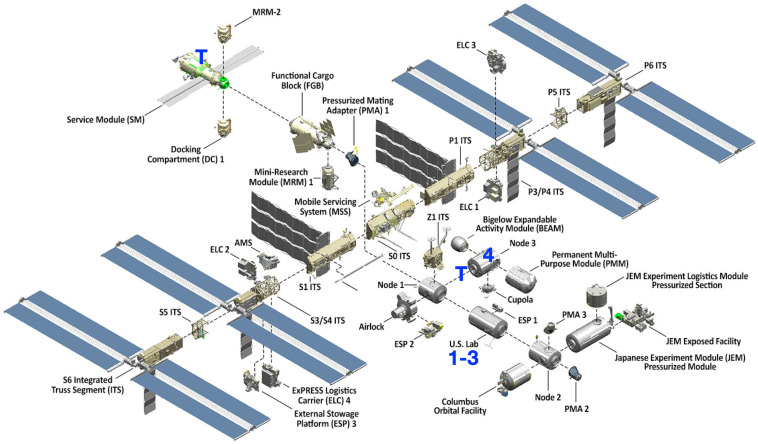
Isolate recovery locations on the International Space Station. Isolates (denoted 1-4) were recovered from the United States Lab and Node 3. Toilets (T) are located in Zvezda (Service Module) and Tranquility (Node 3).

Bacterial isolates from the ISS (Figure 1 and Table 1) were initially recovered on tryptic soy agar, and preliminary identification was performed using a VITEK identification system (bioMérieux, Hazelwood, MO, United States) as previously described (Castro et al., 2004). *E. faecalis* isolates OG1RF, MMH594, V583, and the genome sequences of *E. faecalis* 5952 and JH-1 were made available from the Gilmore lab strain collection. All additional available whole genomes of *E. faecalis* (N = 44) were obtained from the National Center for Biotechnology Information (NCBI).

**TABLE 1:** Summary of genomic characterization of ISS and control strains of *Enterococcus faecalis*.

Isolate (Accession Number)	Sample Type	Location	Collection Date	Expedition(Duration in days)	Genome size (Mb)	No. Unique Genes	MLST	Predicted Intact Phage	Plasmids	Pathogen Score(%)
ISS_1 (CP046113)	Air	United States LAB	7/15/2009	20 (137)	2.65*	11	875**	No	No	84.4
ISS_2 (CP046112)	Surface	United States LAB	4/15/11	27 (70)	2.93*	39	30	3	No	85.4
ISS_3 (CP046111)	Surface	United States LAB	4/15/11	27 (70)	2.94*	39	30	3	No	85.4
ISS_4 (CP0461108-10)	Air	Node 3	10/23/13	37 (61)	2.91*	25	40	1	2	85.4
OG1RF^‡^	Oral	NA	≤ 1975	NA	2.74	61	1	No	No	81.6
MMH594^‡^	Blood	NA	1985	NA	3.25	52	6	1	3	82.9
V583^‡^	Blood	NA	1987	NA	3.36	20	6	1	3	82.9

* Results of hybrid assemblies (See Supplementary Information).

** Single nucleotide variant of the nearest ST.

*^‡^McBride et al. (2007).*

*NA: Not applicable.*

Except where described below, cultures of *E. faecalis* OG1RF, MMH594, V583, and the ISS isolates (Table 1) were grown aerobically with shaking (250 RPM) at 37°C in brain heart infusion (BHI; Difco^TM^, cat. no: 237500) broth. Growth kinetics for each isolate was assessed using a Synergy 2 microplate reader (Bio-Tek Instruments Inc.), measuring optical density at 620 nm. Minimum inhibitory concentration (MIC) for various antibiotics was evaluated in both Mueller-Hinton (MH) broth or in BHI, as further detailed below. For the desiccation experiments, isolates were grown overnight in a chemically defined medium (CDM; Hussain et al., 1991) and plated onto M9 (Difco^TM^, cat. no: 237500) agar plates amended with 1.0% glucose. To perform the *C. elegans* pathogenicity assays, cultures of *Escherichia coli* OP50 were prepared on nematode growth media (NGM) agar plates as previously described (Powell and Ausubel, 2008).

### Genome Sequencing of ISS Strains

For preparation of DNA, isolates were grown overnight in BHI broth. Cells were lysed using lysozyme (50 μg mL^–1^) and mutanolysin (2500 U mL^–1^), and the total DNA was isolated using the DNeasy Blood and Tissue Kit (Qiagen©, cat. no: 69504). Purified DNA was used for preparing libraries, which were then sequenced on Illumina HiSeq (250 nucleotide paired end reads) as previously described (Supplementary Table S1 in Lebreton et al., 2017).

Additionally, a MinION (Oxford Nanopore Technologies©; ONT) library was prepared using the “one-pot” barcoding protocol, as developed by Josh Quick and the Loman Lab^1^. For each isolate, 24 μL of AMPure XP (Beckman Coulter^®^ Life Sciences cat. no.: A63880) eluate was used as input for library preparation. This was incubated at room temperature (RT) for 5 min, 65°C for 5 min, then placed on ice for 30 s. Samples were barcoded using the Rapid Barcoding Kit (ONT cat. no.: SQK-RBK001) and each was incubated at RT for 10 min, 70°C for 5 min, then placed on ice. The reaction product was then pooled with other samples (*N* = 4 total) in a clean 1.5 mL low-bind Eppendorf tube, before addition of 26.75 μl AMPure XP beads per sample. This was incubated at RT for 5 min, placed on the magnet rack until clear, and the supernatant removed. The beads were washed with 200 μl 70% ethanol, incubated for 30 s, and the supernatant removed (2X) before spinning down and removal of the residual 70% ethanol. After air drying for 1 min, the beads were resuspended in 31 μl elution buffer (EB, 10 mM Tris–HCl pH 8), incubated off the magnet rack for 5 min, returned to the rack. DNA was quantified by fluorescence (ThermoFisher Scientific© Qubit 3.0 with Qubit dsDNA HS Assay Kit; cat. no.: Q32854) and the sequencing adaptors were ligated (ONT cat. no.: SQK-LSK109) following the manufacturer’s protocols. This tube was incubated at RT for 10 min, 45.5 μl AMPure XP beads added, incubated for another 5 min, placed on magnet rack until clear, and the supernatant removed. Next, 150 μl ABB was added and the beads resuspended by flicking, before placing the tube back on the magnet rack until clear. The supernatant was removed and the ABB wash repeated before spinning down the tube and removing the residual supernatant. Next, 12 ul EB was added and the bead resuspended by flicking before incubation at RT for 5 min. The tube was placed again on the magnet rack until clear, and the elution library was loaded on to an ONT MinION flow cell (R9.4.1) according to the manufacturer’s protocols and sequenced using MinKNOW (v1.11.5) with live basecalling (Supplementary Table S2). Output fastq files were concatenated and adaptors were removed and debarcoded with PoreChop (v0.2.3^2^). Reads were quality filtered (≥ Q9) using NanoFilt (v2.2.0), and NanoPlot (v1.13.0) was then used to characterize sequencing datasets (De Coster et al., 2018).

### Genome Assembly

Unicycler (v0.4.4) was used for long-read and hybrid assembly using default parameters, with the initial long-read assembly specified via the –existing_long_read_assembly flag for hybrid assembly (Wick et al., 2017). BWA (v0.7.17-r1188 with -x ont2d flag; docker container alexcoppe/bwa:latest) was used to map long reads onto the hybrid assembly to inspect assemblies and verify lack of evidence of mis-assemblies based on coverage. CheckM (v1.0.7) was used to evaluate completeness of final hybrid assemblies described in Table 1 (Parks et al., 2015).

### Pangenomic and Phylogenetic Analysis

**FIGURE 2:**
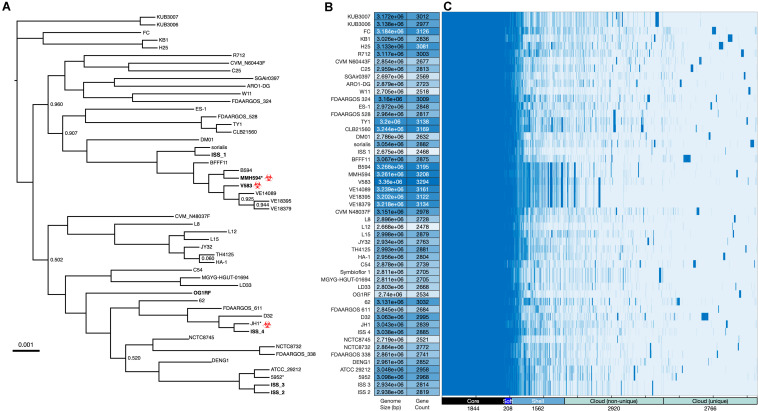
Whole genome analysis of *Enterococcus faecalis*. **(A)** Phylogenetic analysis of *E. faecalis* strains was determined based on FastTree generalized-time-reversible (GTR) analysis of SNPs present in the 1,844 core genes as determined by Roary (Page et al., 2015) and visualized with IcyTree (Vaughan, 2017). The alignment, when corrected for gaps and ambiguous bases, was 1.69 Mb. Branch support was 100% except as indicated. Isolates used in this study are indicated in **bold**. Asterisk (*) denotes an unfinished genome assembly (contigs). Clinical isolates that are phylogenetic neighbors of the ISS isolates are tagged with the biohazard symbol. **(B)** Genome size and gene count, the latter as estimated by Prokka (Seemann, 2014). **(C)** Gene presence/absence as estimated by Roary for the total of 9300 genes identified across the 51 genomes. A histogram of these data is provided as Supplementary Figure S1.

Genomes were annotated using Prokka (v.1.14.6; docker container quay.io/biocontainers/prokka:1.14.6–pl526_0 Seemann, 2014), and the pangenome analysis was performed using Roary (v3.12.0; docker container staphb/roary:3.12.0 Page et al., 2015). Genes that were present in a single isolate were defined as unique genes and, for strains utilized in this study, their identities were further examined by BLASTn (Altschul et al., 1990) for comparison to the NCBI non-redundant database. Based on DNA sequence comparison, an *E. faecalis* phylogenetic tree (Figure 2A) was constructed from the Roary-determined core gene alignment (1.69 Mb) using FastTree (docker container staphb/fasttree:2.1.11) using the General Time Invariant (GTR) model, and visualized using IcyTree (Vaughan, 2017). For a list of all genomes and gene presence/absence data see Supplementary Data File S1.

### Comparative Genomic Analysis

Multilocus sequence typing (MLST) was performed by evaluating seven *E. faecalis* genes (*aroE*, *gdh*, *gki*, *gyd*, *pstS*, *xpt*, and *yqiL*) and sequence types (STs) assigned based on the alleles present in each genome (Larsen et al., 2012). The potential presence of plasmids was assessed using PlasmidFinder (Carattoli et al., 2014). VirFinder was used to detect potential *E. faecalis* virulence factor genes (Joensen et al., 2014), and PathogenFinder provided a predictive score for each strain’s potential pathogenicity for humans (Cosentino et al., 2013). Additionally, the web server PHAge Search Tool – Enhanced Release (PHASTER) was used to detect the presence of phage sequences within bacterial genomic data (Arndt et al., 2016), and the Comprehensive Antibiotic Resistance Database (CARD) was used to identify genes potentially associated with antimicrobial resistance (Jia et al., 2016). The presence of CRISPR (clustered regularly interspaced short palindromic repeats) arrays and Cas (CRISPR associated proteins) was detected using the online tool, CRISPRCasFinder (Couvin et al., 2018). Capsule type polymorphisms were determined as described by McBride et al. (2007). Briefly the presence, or absence, of genes in the locus (EF2485 to EF2495) were verified in the genomes of each isolate. CPS type 1 strains are defined as isolates with *cpsA* and *B*, and *hcp1* (EF2484), while CPS type 2 strains consist of the full *cps* locus and *hcp1* (EF2484 to EF2495). CPS type 5 strains are similar to the type 2 gene profile, but do not contain *cpsF* (EF0090).

### Minimum Inhibitory Concentration of Antibiotics

Antibiotic MIC was determined by broth microdilution as described (CLSI, 2017). Briefly, overnight cultures of isolates were diluted and 10^4^ cells in MH broth were inoculated into each well of 96 well plates containing serial 2-fold dilutions of antibiotics. Maximum concentration tested: penicillin (10 μg mL^–1^), amoxicillin (10 μg mL^–1^), ampicillin (10 μg mL^–1^), oxacillin (256 μg mL^–1^), erythromycin (50 μg mL^–1^), and tetracycline (64 μg mL^–1^). This procedure was repeated in BHI (McBride et al., 2007), and isolates were additionally evaluated for high level aminoglycoside resistance (HLGR) with: gentamicin, streptomycin, and kanamycin (each at a maximum concentration of 2 mg mL^–1^). Plates were incubated at 37°C and growth was monitored at 24 and 48 h. Wells containing the lowest antibiotic concentration lacking growth after 48 h were designated the MIC for the respective antibiotics.

### Desiccation Survival

Survival to desiccation was assessed as previously described (Lebreton et al., 2017; Bryan et al., 2019) with the following modifications. *E. faecalis* isolates were harvested from stationary phase by centrifugation, and washed twice with sterile (autoclaved, 0.22 μm filtered) water (sH_2_O). Prior to desiccation, the concentrated cells were resuspended in CDM, and a 10 μL sample was track diluted (Jett et al., 1997) onto M9 medium (M9; Amresco^®^, cat. no.: J863-500G) amended with 0.1% glucose agar, and incubated at 37°C. After ∼24 h, the number of CFUs recovered from 10 μL track dilutions prepared according to Jett et al. (1997) were used to determine the original concentration of cells mL^–1^ (*t* = −1). Aliquots of cell suspensions (50 μL) were spotted onto autoclaved coverslips, and placed into the desiccation chamber containing Drierite^®^ (≥ 98% CaSO_4_, W. A. Hammond Drierite Company, Ltd., cat. no.: 778-18-9) until the liquid evaporated (*t* = *0*, 24 h). The relative humidity (RH) in the chamber was monitored using a digital hygrometer, and maintained between 30-40% by adding additional Drierite^®^. Three replicates were prepared for each isolate at each timepoint. The dried cells were removed from the coverslips by rinsing and resuspending the material with a pipet into 1 mL of sH_2_O. To ensure the samples were homogenously resuspended, rehydrated cells were incubated at 37°C for 1 h while shaking (225 RPM), and vortexed for 15 minutes. The cells were then serially diluted into sH_2_O and 10 μL track dilutions were plated onto M9 agar. At the *t* = 0 time point, the number of CFUs recovered from the substrate was designated as the starting population size that survived the initial drying on the coverslip (*N*_0_). Subsequent samples were removed from the desiccation chamber every three days and rehydrated as described above to determine the number of surviving CFUs over time (*N*). The surviving fraction of cells was determined from the ratio of *N/N_0_*. Each time point was evaluated when *N* was ≥ 30 CFU.

### Pathogenicity of *E. faecalis* isolates in a *C. elegans* model

Nematode killing assays were performed as previously described (Yuen and Ausubel, 2018). Briefly, *C. elegans fer-15;fem-1* (CF512) worms were grown on NGM inoculated with *E. coli* strain OP50 and incubated at 15°C until they reached the L4 stage. Approximately 30–40 of the L4 stage worms were then transferred to BHI agar plates (amended with kanamycin, 10 μg mL^–1^) inoculated with 100 μL aliquots of each log phase *E. faecalis* isolate. The plates were incubated at 25°C and worms were examined for viability. Worms that did not exhibit movement nor respond to physical touch were scored as dead and removed from the plate. Moving worms were scored as alive and also counted.

### Statistical Analyses

Statistical analyses for the desiccation experiments were performed using JMP^®^ Pro v.14.3 (JMP^®^, 2019). Prior to evaluating the data for significant differences, the data were assessed for equal variances. Subsequent one-way ANOVAs were performed to determine the significant differences of desiccation survival at different timepoints and among individual isolates. *Post hoc* comparisons for time were performed using parametric comparisons with a control using Dunnett’s method (*N* = 18 for each isolate, with the exception of *t* = 12 d where *N* = 17). The mean *N/N_0_* for each isolate at *t* = 21 d was evaluated using the non-parametric comparison with a control performed following the Steel method (*N* = 3 for each isolate). Individual comparisons for survival at two timepoints were performed using a homoscedastic Student’s *t-* test.

The survival of *C. elegans* on each *E. faecalis* isolate was performed in triplicate. A Kaplan-Meier log rank analysis was performed to evaluate the survival curves using OASIS-2 (Han et al., 2016) as previously described Yuen (Yuen and Ausubel, 2018). The data were determined to follow the normal distribution using the Shapiro-Wilk test (Han et al., 2016), and pairwise comparisons with Bonferroni-corrected *p*-values < 0.05 were considered significant. Data are reported as the average and the standard error (± SE) of the number of worms scored, and is reported in Table 2.

**TABLE 2:** Analysis of the survival of *Caenorhabditis elegans* infected with the ISS and control strains of *Enterococcus faecalis*.

Isolate	N*	Restricted Mean			LT_50_	Pairwise Comparisons**
		
		Days	SE	95% C.I.		
ISS_1	37.0	9.00	0.66	7.44–10.0	8.13	BC
ISS_2	33.0	13.0	0.53	12.5–14.5	12.9	AB
ISS_3	29.0	13.0	0.59	12.0–14.3	13.1	ABC
ISS_4	35.0	10.0	0.70	9.06–11.8	10.4	BC
OG1RF	42.0	10.0	0.48	9.5–11.3	10.0	BC
MMH594	35.0	9.00	0.50	8.4–10.3	8.88	BC
V583	34.0	11.0	0.91	8.71–12.3	9.00	ABC

The data are reported as the restricted mean (± SE) of three replicates, with the exception of ISS_1 (*N* = 2 at t = 9 *d*). * Starting number of worms. ** Pairwise analysis of each Kaplan-Meier survival curve using the log-rank test (Han et al., 2016). Those that were not significantly different from each other are indicated with a capital letter, with A being the least pathogenic (*p* > 0.05).

## Results

### Isolates Recovered From the ISS

The *E. faecalis* isolates recovered from air samples (ISS_1 and ISS_4) were both collected mid-module. ISS_1 was isolated from air samples collected in the United States Lab in 2009, and ISS_4 was recovered from the air in Node 3 in 2013 (Figure 1). ISS_2 and ISS_3, were two isolates recovered in 2011 from the same location, United States Lab handrail surfaces, approximately 14 feet apart. One was collected from the aft end of the module (at the interface with Node 1), and the other was recovered mid-module. The United States Lab was launched in 2001, and houses the various science payloads conducting research onboard the ISS (Singh et al., 2018b). Node 3, launched in 2010, and houses the air revitalization and water recovery systems, as well as amenities for crew hygiene, the restrooms, and exercise devices (Singh et al., 2018b). With preliminary identification based solely on the VITEK identification system, the first task for the isolate characterization was to create a fully sequenced genome for each isolate (Table 1).

### Pangenome Analysis

With the complete genomes for each isolate assembled, several web-based tools were utilized for genomic characterization. First, seven gene loci were evaluated for their respective MLST using the web-based MLST server (Table 1; Larsen et al., 2012). ISS_1 was determined to be a single locus variant of sequence type (ST) 875, with a single nucleotide polymorphism (SNP) in the 583 bases of *pstS*. Isolates ISS_2 and ISS_3 were identified as ST30. ISS_4 was classified as ST40, a commonly recovered ST and a member of one of the largest clonal clusters, CC40 (McBride et al., 2007). A clonal cluster is defined as a ST consisting of three or more isolates (McBride et al., 2007). CC40 is comprised of ST40 and ST114, many of which were isolated from clinical sources (McBride et al., 2007).

Based on the ST data described above, representative type strains were chosen for each as a comparator for each ISS ST (McBride et al., 2007), and a core genome of 1,844 genes shared by all 51 strains was defined (Page et al., 2015). An alignment of the core genome was then used to construct the phylogenetic tree (Figure 2A). Phylogenetic proximity of the selected type strains to the ISS isolates validated the selection based on ST. Genome size and gene count (Figure 2B) show patterns of genomic expansion and loss within clades; for example, within the clade containing V583 a general pattern of larger genome size and higher gene count is evident, yet nearby ISS_1 has a more streamlined genome in comparison to its neighbors. Genes are classified into core, soft core, shell, and cloud on the basis of the number of isolates sharing each gene (Figure 2C; Supplementary Figure S1) and the Roary-estimated patterns of gene presence and absence highlight potential patterns of gene loss or gain (Supplementary Data File S1).

Unique chromosomal genes, defined as those genes that were present in a single isolate, were non-uniformly distributed across each isolate genome (Supplementary Data File S1). ISS_1 (Accession no.: CP046113) contained the fewest number of unique genes (11 genes), 9 of which were designated hypothetical by Prokka (Seemann, 2014) yet had homology with other *E. faecalis* genomes on the basis of BLASTn searches. Seven of the 11 unique genes formed a contiguous region suggestive of an operon, which contained two genes with known annotations, *mazG*, which encodes a nucleoside triphosphate pyrophosphohydrolase involved in survival under nutrient stress, and another that encodes an LPxTG-motif protein cell wall anchor domain protein. Neither ISS_2 (Accession no.: CP046112) nor ISS_3 (Accession no.: CP046111) contained individually unique genes. Consistent with this finding, analysis of the core alignment revealed that isolates ISS_2 and ISS_3 differed by only 14 bases. However, these two isolates shared 39 genes that were not present in any of the other strains analyzed, of which 30 were classified as hypothetical. ISS_4 (Accession no.: CP046108-110) had 25 unique genes, 16 of which were located on a plasmid (pTEF2); no unique genes were found on the second (pAD1) plasmid. Based on BLASTn analysis (Supplementary Figure S2), the majority of genes unique to the ISS strains are closely related to genes present in *Enterococcus* species, with all non-*Enterococcus* genera restricted to *Lactococcus*, *Lactobacillus*, and, with lower identity and query coverage, *Listeria* (Supplementary Figure S3).

Following the designations of McBride et al. (2007), the capsule type for each ISS strain was determined. All three known capsule type polymorphisms were detected from the ISS strains. ISS_1 contained the full *cps* locus (EF2485 to EF2495) and *hcp1* (EF2484), and similar to MMH594 and V583, was designated CPS type 2. Isolates ISS_2 and ISS_3 contained the *cps* locus and *hcp1*, but lacked *cpsF* (EF0090). In agreement with previous analysis of two additional ST30 isolates (McBride et al., 2007), these strains were classified as CPS type 5. The strain ISS_4 contained only *cpsA* (EF0095), *cpsB* (EF0094), and *hcp1*, and like the other isolates of CC40 and OG1RF, was classified as CPS type 1 (McBride et al., 2007).

### Assessment of Mobile Elements and Defense Systems

The Comprehensive Antibiotic Resistance Database (CARD) database identified the tetracycline resistance gene, *tetW/N/W*, in isolates ISS_2, ISS_3, and ISS_4, which was not present in OG1RF (Jia et al., 2016). The ISS *tetW/N/W* shared 69.3 (ISS_2 and ISS_3) and 69.1% (ISS_4) sequence homology with the reference sequence (Accession no.: ARO:3004442). The reference gene has been detected in 2.70% of the available *E. faecalis* isolates surveyed (Jia et al., 2016). A standard nucleotide BLAST search confirmed the *tetW/N/W* genes present in ISS_2 and ISS_3 shared 100% sequence identity to each other, and differed by three SNPs to the *tetW/N/W* gene present in ISS_4 (Altschul et al., 1990).

PHASTER identified the presence of intact phage in isolates ISS_2, ISS_3, and ISS_4 (Supplementary Table S3; Arndt et al., 2016). ISS_2 and ISS_3 each contained three intact phage nucleotide sequences. The CC40 member ISS_4 contained a single intact phage region. No intact phage were detected in either ISS_1 or OG1RF. Thirty two (82%) of the 39 genes shared only by ISS_2 and ISS_3 were associated with intact or incomplete phage (Supplementary Data File S1). Plasmid-born genomic elements were only detected in ISS_4 using the tool PlasmidFinder (Table 1; Carattoli et al., 2014). Two plasmids were determined to share 96.7% sequence identity, and 99% coverage, to the V583 plasmid pTEF2 (accession number: AE016831; Paulsen et al., 2003), and 96.0% identity, with 100% coverage, to the pheromone responsive plasmid pAD1 (accession number: L01794; Ehrenfeld and Clewell, 1987). Notably, evidence for *Lactobacillus prophage* Lj928 was identified for ISS_2, ISS_3, ISS_4, and V583 (Supplementary Table S3). Similarly, evidence for *Enterococcus phage* phiFL4A was identified for ISS_2, ISS_3, ISS_4, and MMH594 (Supplementary Table S3). Overall, mobile elements explained a majority (65%) of the unique genes identified in the ISS isolates. High confidence CRISPR-Cas systems were detected in isolates ISS_1, ISS_4, and OG1RF (Supplementary Table S4). CRISPR loci without the Cas genes were detected in the genomes of each of the ISS isolates and OG1RF (Supplementary Table S4).

### Genome-Based Assessment of the Potential for Pathogenicity

The PathogenFinder server scored each of the ISS isolates and the reference strains as potential human pathogens (Table 1; Cosentino et al., 2013). The commensal strain OG1RF had the lowest pathogen score of 81.6%. With pathogen scores > 84%, all of the ISS strains had a higher assigned probability to be pathogenic than the clinical isolates, MMH594 and V583 (each at 82.9%).

By comparing the genomic content of the isolates to known *E. faecalis* genes associated with disease, a second predictor of pathogenicity could be compiled (Table 3; Joensen et al., 2014). The genomes of the clinical isolates contained the highest proportion of predicted virulence factors (18/20), while ISS_2 and ISS_3 contained the least (13/20). ISS_1 and OG1RF both contained 15/20 virulence factors, and the genome ISS_4 was positive for 16/20. The clinical isolates and ISS_4 were the only strains to contain *agg*, encoding for the *E. faecalis* aggregation substance involved in biofilm formation, plasmid transfer, and increased virulence in endocarditis models (Bhatty et al., 2015).

**TABLE 3:** The presence, and sequence identity (%), of known virulence factors of *Enterococcus faecalis* present in the ISS and reference genomes (Joensen et al., 2014).

Virulence factor	Identity (%)						
	
	ISS_1	ISS_2	ISS_3	ISS_4	OG1RF	V583	MMH594
ElrA	99.5	99.72	99.72	100	100	100	100
SrtA	100	100	100	100	100	100	100
*ace*	98.5	97.71	97.71	96.9	97.5	100	
*agg*				95.6		100, 96.8*	100
cCF10	99.8	99.9, 99.9*	99.9	99.8, 99.8*	100, 100*		
cOB1	100	100	100	99.9	100	100	100
*cad*	99.9	100	100	99.8	100	100	100
*camE*	100	99.6, 99.6*	99.6	100, 100*	100	100	100
*cylA*							99.8, 99.8*
*cylL*						100	100
*cylM*						100	100
*ebpA*	100	100	100	100	100	100	100
*ebpB*	100	99.8	99.8			100	100
*ebpC*	100			100	100	100	100
*efaAfs*	100	99.9	99.9	100	100	100	100
*fsrB*	99.9			100	100	100	100
*gelE*	99.4	99.9	99.9	100	100, 100*	100	100
*hylA*				99.9	100	100	99.7
*hylB*	99.9	99.5	99.5	99.6	100	100	100
*tpx*	100	100	100	100	100	100	100

* Multiple copies were detected.

MMH594 was the only isolate with *cylA*, one of the genes responsible for the production of cytolysin. Neither OG1RF nor the ISS isolates carried any of the genes in the cytolysin pathway reviewed by Coburn and Gilmore (2003).

### Minimum Inhibitory Concentration of Antibiotics

Dilution concentration broth assays were used to determine the MIC for various antibiotics. Optical density measurements revealed there were no significant differences in the growth kinetics between the ISS isolates and the reference strains. Strains were first evaluated in Mueller-Hinton broth for comparison to clinical laboratory standards for antibiotic resistance of *E. faecalis* ATCC^®^ 29212 (CLSI, 2017), and in the commonly used growth media BHI to determine MICs relative to the commensal isolate OG1RF. In MH broth, all strains tested exceeded the resistance levels set forth by ≥ 2-fold for amoxicillin, ampicillin, oxacillin, gentamycin, and kanamycin (CLSI, 2017). ISS_2, ISS_3, and ISS_4 were also categorized as resistant to tetracycline. When evaluated in BHI, all ISS strains exhibited MICs equivalent to OG1RF with the exceptions of oxacillin and tetracycline (Table 4). All ISS strains showed ≥ 4-fold more resistance to oxacillin. Isolates ISS_2, ISS_3, and ISS_4, each carrying a *tetW/N/W* gene, displayed an MIC of 64 μg mL^–1^. These isolates were 4-fold more resistant to tetracycline than OG1RF (MIC of 16 μg mL^–1^), and displayed resistance levels equivalent to the clinical isolates, MMH594 and V583. Unlike the clinical isolates, the ISS strains did not exhibit HLGR.

**TABLE 4:** Minimum inhibitory concentration of antibiotics evaluated in BHI.

Isolate	Antibiotic MIC (μg mL^–1^)								
	
	Penicillin	Amoxicillin	Ampicillin	Oxacillin	Erythromycin	Tetracycline	Gentamycin	Streptomycin	Kanamycin
ISS-1	10.0	> 10.0	2.50	256	1.56	16.0	125	500	500
ISS-2	5.00	> 10.0	2.50	128	1.56	64.0	125	500	500
ISS-3	5.00	> 10.0	2.50	128	1.56	64.0	125	500	500
ISS-4	10.0	> 10.0	2.50	128	1.56	> 64.0	62.5	500	250
OG1RF	5.00	> 10.0	2.50	32.0	1.56	16.0	125	500	500
MMH594	10.0	> 10.0	5.00	128	> 50.0	64.0	> 2.00 × 10^3^	1.00 × 10^3^	> 2.00 × 10^3^
V583	2.50	10.0	2.50	256	> 50.0	64.0	> 2.00 × 10^3^	500	> 2.00 × 10^3^

### Desiccation Tolerance of *E. faecalis* Strains

**FIGURE 3:**
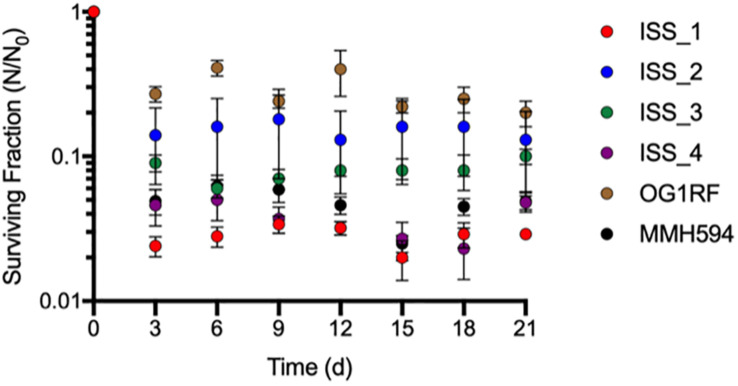
Desiccation survival of *Enterococcus faecalis* strains from the ISS and the control strains, OG1RF and MMH594. The data are presented as the average (± SD) of triplicate measurements. Some error bars have been obscured by the data points.

During the initial 24 h dry down period, the RH in the desiccation chamber spiked to 56%. For the remainder of the experiment, the RH was maintained between 30-40% by adding additional Drierite^®^. The concentration of cells surviving the drying process (*N*_0_) ranged from 1.2 (± 0.044) x 10^10^ to 5.5 (± 0.091) x 10^7^ CFU for ISS-1 and OG1RF, respectively. The average number of survivors (*N*) after three weeks of desiccation ranged from 0.11 (± 0.024) to 8.5 (± 0.79) x 10^8^ CFU for OG1RF and MMH594, respectively. OG1RF demonstrated the greatest loss in viability during dry down with a 2.1-log reduction, and ISS-2 was the only isolate that did not experience a significant loss in viability between *t* = 0 and *t* = 3 d (*p* = 0.11). There was no significant difference in *N/N_0_* at any of the timepoints evaluated (*p* = 0.98; Figure 3). When the variances of *N/N_0_* at t = 21 d for each isolate were determined to be significantly different from each other (*F* = 58, *p* < 0.001), a non-parametric comparison was performed with OG1RF chosen as the control using the Steel method. There was no significant difference in *N/N_0_* at *t* = 21 d among the isolates tested when compared to OG1RF (Figure 3).

### Pathogenicity of Isolates in a *C. elegans* Model

**FIGURE 4:**
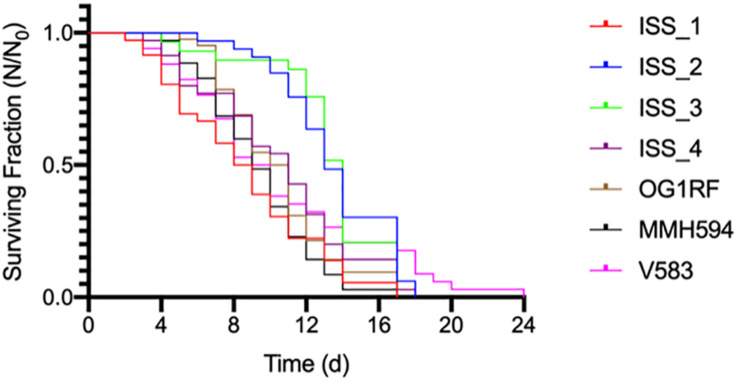
The average survival of *Caenorhabditis elegans* exposed to ISS and control strains of *Enterococcus faecalis.*

The survival data (Figure 4) were determined to follow the normal distribution using the Shapiro-Wilk test (Han et al., 2016). With the exception of ISS_1, all data are reported as the mean (± SE) of three replicates. For ISS_1, one sample became contaminated with fungus on day 9, and all subsequent data are reported as *N* = 2. *C. elegans* was least susceptible to strains ISS_2, ISS_3, and V583 with mean survival times of 13 (± 0.53), 13 (± 0.59), and 11 (± 0.91) days, respectively (Table 2). While the time for 50% of the worm population to die (LT_50_; Garsin et al., 2001) for these isolates ranged from 9 days (V583) to 14 (ISS_3), the total lifespan of worms challenged with these isolates were not significantly different from each other. For isolates ISS_2 and ISS_3, all worms were deceased by day 18 and 17, respectively. While 90% of worms fed V583 were dead by day 18, some survivors persisted until day 24. Additionally, the lifespan of worms infected with ISS_3 was also statistically similar to those challenged with ISS_4. With a 31% decrease in the mean lifespan, worms infected with ISS_1 were significantly more susceptible than isolates fed ISS_2 or ISS_3 (*p* < 0.001). Based on the survival curves of worms fed ISS_1, ISS_4, OG1RF, MMH594, and V583 there were no significant differences in the pathogenicity profiles for these isolates (*p* > 0.05).

## Discussion

While multiple studies have detected the presence of a wide range of opportunistic human pathogens onboard the ISS, the consensus was that additional data was needed to determine if these microorganisms posed an actual threat to crew health (Schiwon et al., 2013; Be et al., 2017; Lang et al., 2017; Singh et al., 2018a,b; Mora et al., 2019; [Bibr B55][Bibr B58]; Urbaniak et al., 2019). Here, we expanded upon previous genomic analyses in an effort to provide a framework for the determination of the pathogenic potential of *E. faecalis* isolates. For the comparative analyses, the commensal type strain OG1RF and the clinical isolates, MMH594 and V583, were chosen because of their extensive genotypic (Shankar et al., 2002; Paulsen et al., 2003; McBride et al., 2007; Bourgogne et al., 2008) and phenotypic characterization, specifically in regards to *C. elegans* pathogenesis (Garsin et al., 2001; Yuen and Ausubel, 2018). In addition, the *E. faecalis* genomes of strains 5952 and JH-1 were chosen as representative ST strains of the ISS isolates ISS_2 and ISS_3, and ISS_4, respectively.

While the amount of information that can be gleaned from a complete genome is large, the current methodologies (Singh et al., 2018a) are insufficient for the accurate assessment of pathogenicity for *E. faecalis* strains. The scores from the tool, PathogenFinder (Cosentino et al., 2013), predicted each of the ISS isolates as possessing > 84% probability of being pathogenic to humans, placing them above known clinical isolates MMH594 and V583 (Table 1). Another tool related to scoring pathogenicity, VirFinder, identified twenty virulence factors among the ISS strains, but fifteen were present in the commensal isolate OG1RF as well (Table 3). We cautiously interpret this to mean that ISS isolates do share some of the potentially virulence-related properties found in clinical isolates, but many of these are also shared by prototype commensal strains of *E. faecalis*. Further validation for these predictive algorithms applied to enterococci is needed in order to make more accurate predictions of which strains could impact human health. Predictions from PathogenFinder or its equivalent can be expected to improve if additional training data at the genus or species level is incorporated.

Because of the difficulty in interpreting potential pathogenicity of the ISS strains based on genome content alone, we tested them directly in the *C. elegans* infection model (Figure 4). Previous studies identified parallels in the pathogenicity profiles of various enterococcal lineages when tested in *C. elegans* (Garsin et al., 2001) and in mice (Bourgogne et al., 2008). In contrast to that report (Garsin et al., 2001), we did not detect significant difference in *C. elegans* survival when exposed to commensal OG1RF, or pathogenic V583 and the cytolytic strain MMH594. *E. faecalis* proliferate in the intestine of *C. elegans* (Garsin et al., 2001) ultimately occluding it (Yuen and Ausubel, 2018). As a result, *C. elegans* infected with *E. faecalis* reach the LT_50_ 33% faster than worms fed a normal diet of *E. coli* OP50 (Garsin et al., 2001). None of the *E. faecalis* strains tested here, including ISS strains, exhibited enhanced *C. elegans* killing (Figure 4). Closely related strains ISS_2 and 3, despite possessing a high computed pathogenicity score, were found to be least able to kill *C. elegans* (Figure 4).

Additionally, there are currently no validated methods to predict phenotypic responses of enterococci to environmental stressors (*e.g*., desiccation, starvation, increased cosmic radiation) based on their genomic content alone. Because each of the ISS isolates were recovered from air and surface samples, we examined them specifically for the ability to survive desiccation at RH relevant to the clinical environment (ASHRAE, 2013). In general, members of the genus *Enterococcus* are more desiccation tolerant than closely related microorganisms (Lebreton et al., 2017). Blaustein et al. (2019) demonstrated that the spaceflight environment onboard the ISS did not select for “extremo-tolerant” strains of *B. cereus* and *S. aureus*. Likewise, we found no significant differences in desiccation tolerance between the ISS *E. faecalis* isolates and their representative type strains (Figure 3). This indicates that their environmental survival in the ISS was attributable to the previously described intrinsically rugged nature of E. *faecalis* (Lebreton et al., 2017). Tirumalai et al. (2018) compared the genomes of the highly resistant *B. safensis* FO-36b and *B. pumilus* SAFR-032 to over 60 reference strains, and also failed to find genomic signatures for their increased environmental resistances (*e.g.*, desiccation, starvation, increased ultraviolet radiation). Rather, it is more likely the regulation of a suite of genes that confers increased resistance as a generalized stress response. In agreement with Blaustein et al. (2019) we conclude that the interior space environment (*e.g.*, microgravity, increased cosmic radiation) selects for intrinsically rugged species of bacteria, such as *E. faecalis*, but as of yet, there is no evidence suggesting selection for enhanced environmental persistence.

In agreement with previous observations (Schiwon et al., 2013; Sobisch et al., 2019), the *E. faecalis* isolates examined here were resistant to multiple antibiotics. While the detection of multidrug resistant bacteria may sound alarming, much of that resistance is intrinsic to all enterococci, and other genetic signatures indicate that these are largely commensal strains (Table 3). However, resistance to tetracycline displayed by ISS_2, ISS_3, and ISS_4 was found to be mediated by an acquired *tetW/N/W* gene, which confers an MIC equivalent to that of clinical isolates (Table 4). In addition to harboring genes that make combating infections difficult, *E. faecalis* strains possess the ability to spread antibiotic resistance to genes to other bacterial species, including the commonly isolated ISS inhabitant *S. aureus* (Sobisch et al., 2019). Although there have been no reports of VRE isolated from onboard the ISS at the time of this publication, it is important to note that both vancomycin resistance genes and *E. faecalis* genomic signatures were detected concurrently from the surface of the dining table during expedition 43 (Singh et al., 2018b). During the same mission, *E. faecalis* was also ranked the second most abundant BSL-2 microorganism in the metagenomic analysis of the entire campaign (Singh et al., 2018b). In fact, Mora et al. (2019) determined *Enterococcus* to be one of the ten indicator genera that distinguished the ISS microbiome from the human and indoor samples of the Home Microbiome Project (Mora et al., 2019).

There were several limitations to the data presented above. The desiccation survival experiments were terminated before any significant decrease in viability was observed. Extending the experiment until no surviving cells remained may have revealed a difference in the survival curves among the strains and provide insight to the length of time *E. faecalis* can remain viable. The lack of experimentally validated computational tools for *E. faecalis* led to conflicting results when comparing the genomes known clinical isolates to commensal strains. In addition, *C. elegans* serves as a rudimentary model system for human infection. However, the challenges presented here provide the foundation for future investigations of *E. faecalis*, on Earth or in space.

As members of the core gut microbiome, *E. faecalis* will continue to be transported to the ISS and the ability to detect pathogenic strains will be beneficial for crew health. Unlike most environmental bacteria (Staley and Konopka, 1985), *E. faecalis* strains are routinely recovered on culture media. Based on the antibiotic resistance profiles presented here, incorporating the above high-level aminoglycosides to the routine onboard culturing activities could serve as the first step toward distinguishing pathogenic from commensal strains of *E. faecalis*. Because of their commonness as survivors on abiotic surfaces, their potential ability to infect, as well as the ability of *E. faecalis* to readily acquire and spread mobile elements conferring antibiotic resistance, continued microbial monitoring and pathogen identification will be important.

Current microbial monitoring of ISS air and surface sampling occurs once a month for the first 90 days of a mission, and decreases to one sample event for every 90 days thereafter (ISS-MORD, 2013). Because these microbial monitoring procedures are culture based, there is an inherent bias toward the small fraction of bacterial species that will form a colony on a plate (Staley and Konopka, 1985). Assessing the total microbial burden, and viable fraction, by combining genomic and culture-based analyses provides a more accurate representation of the ISS microbiome (Be et al., 2017; Mora et al., 2019; Sielaff et al., 2019). However, it is still unclear which, if any, of these microorganisms are relevant to crew health (Schiwon et al., 2013; Be et al., 2017; Lang et al., 2017; Singh et al., 2018a,b; Mora et al., 2019; Sielaff et al., 2019; Sobisch et al., 2019; Urbaniak et al., 2019).

The need for expedient identification of pathogenic microorganisms and their antibiotic resistance profiles is not a challenge unique to the spaceflight environment. Recent advances in rapid diagnostics have helped alleviate the clinical reliance on culture based techniques to identify infectious agents, yet these resources are not always widely available (Maurer et al., 2017). Fortunately, this is not the case for the ISS where the crew have access to Wetlab-2 Cepheid© SmartCycyler that can be used for quantitative PCR (qPCR) analysis of air, surface, water, and clinical samples^3^. Although it is currently configured to detect only a limited number of microbial pathogens, the RAZOR^TM^ EX BioDetection System (Idaho Technology Inc.) is currently being developed for in-flight microbial monitoring and could be modified for a wider range of microbial targets. Another promising technology for pathogen detection is the ONT MinION sequencing device employed above. Castro-Wallace et al. (2017) have previously validated its efficacy in the spacecraft environment. The MinION is currently capable of identifying bacterial species in near real time from metagenomic data (Sanderson et al., 2018) or isolated samples (Gargis et al., 2019) and has been used for the first ever off-Earth identification of bacteria cultured and sequenced in flight (Burton et al., 2020).

Due to the inability for the existing tools to distinguish pathogenic from commensal strains of *E. faecalis*, we make the following recommendations: Rather than continuing to wait for sample return and analysis using the current unvalidated databases, we suggest performing an initial screen for HLGR. Then, any isolates exhibiting HLGR should be sequenced using the MinION and evaluated for the presence of the type 2 capsule (McBride et al., 2007), the vancomycin resistance operon observed in V583 (Paulsen et al., 2003), components of the pathogenicity island (*e.g*., cytolysin production loci and the enterococcal surface protein, Esp), and the absence of functional CRISPR-Cas arrays (Van Tyne et al., 2019). This information would then provide near real-time information for the crew regarding the need for additional decontamination procedures. These advancements would not be limited to *E. faecalis* isolates, however more accurate genomic targets for additional pathogen detection will need to be experimentally validated (Chiu and Miller, 2019). With the development of such targets, multiple pathogens (including bacteria, fungi, and viruses) can quickly be identified within a single sample (Miller et al., 2019).

## Data Availability Statement

The datasets generated for this study can be found in the NCBI BioProject website (https://www.ncbi.nlm.nih.gov/bioproject) using the BioProject ID: PRJNA587161 and the accession numbers SAMN13182395–SAMN13182398.

## Author Contributions

NB designed the experiments, performed the research, analyzed the data, and wrote the manuscript. CC assembled the genomes and advised on bioinformatic analysis and experimental design. MG advised on experimental design. FL advised on bioinformatic analysis and experimental design. GR and MZ advised and reviewed the manuscript. All authors contributed to the manuscript.

## Conflict of Interest

The authors declare that the research was conducted in the absence of any commercial or financial relationships that could be construed as a potential conflict of interest.

## Footnotes

1 https://lab.loman.net/protocols/

2 https://github.com/rrwick/Porechop

3 https://www.nasa.gov/spacebio/microbiology/hardware

## Supplementary Material

Supplementary Figure 1 Gene membership of the 51 *Enterococcus faecalis* isolate genomes analyzed. Counts and categories as estimated by Roary analysis of presence/absence as described in the methods.

Supplementary Figure 2 Sequence similarity of *Enterococcus faecalis* genes unique to ISS_1, ISS_2/ISS_3, and ISS_4. Query coverage and percentage sequence identity from BLASTn searches across all isolates (upper left) and for each isolate, respectively. Because ISS_2 and ISS_3 have no individually unique genes, and differ in their core gene alignment by only 14 bases, but have genes that are not represented in any of the other 49 genomes studied, ISS_3 was excluded from this analysis. Supplementary Data File 1 contains details of each BLASTn hit.

Supplementary Figure 3 Sequence similarity of *Enterococcus faecalis* genes unique to ISS_1, ISS_2/ISS_3, and ISS_4 segmented by the genus of the hit subject. Query coverage and percentage sequence identity from BLASTn searches are as in Supplementary Figure S2 (upper left) after eliminating all hits associated with *Enterococcus* (upper left) and further segmenting by genus (there were only three remaining genera).

Supplementary Table 1 Illumina HiSeq short read coverage estimates.

Supplementary Table 2 Nanopore long read coverage estimates.

Supplementary Table 3 Predicted intact phage content as determined by PHASTER for ISS and control *E. faecalis* isolates (Arndt et al., 2016).

Supplementary Table 4 CRISPR-Cas system detection in the genomes of ISS and reference strains (Couvin et al., 2018).

Supplementary Data File 1 Excel file listing all genomes used in the project, the detected presence/absence of each annotated gene for all genomes, and BLASTn hits for genes unique to the ISS isolates.
