# Supplementary material for: Genomic and Functional Characterization of Enterococcus faecalis Isolates Recovered From the International Space Station and Their Potential for Pathogenicity
Source: Front Microbiol. 2021 Jan 11;11:515319. doi: 10.3389/fmicb.2020.515319 (PMC7829349; doi:10.3389/fmicb.2020.515319)
Supplement: Supplementary Figure 1 — Gene membership of the 51 Enterococcus faecalis isolate genomes analyzed. Counts and categories as estimated by Roary analysis of presence/absence as described in the methods. [file Data_Sheet_2.PDF]

Supplementary Material

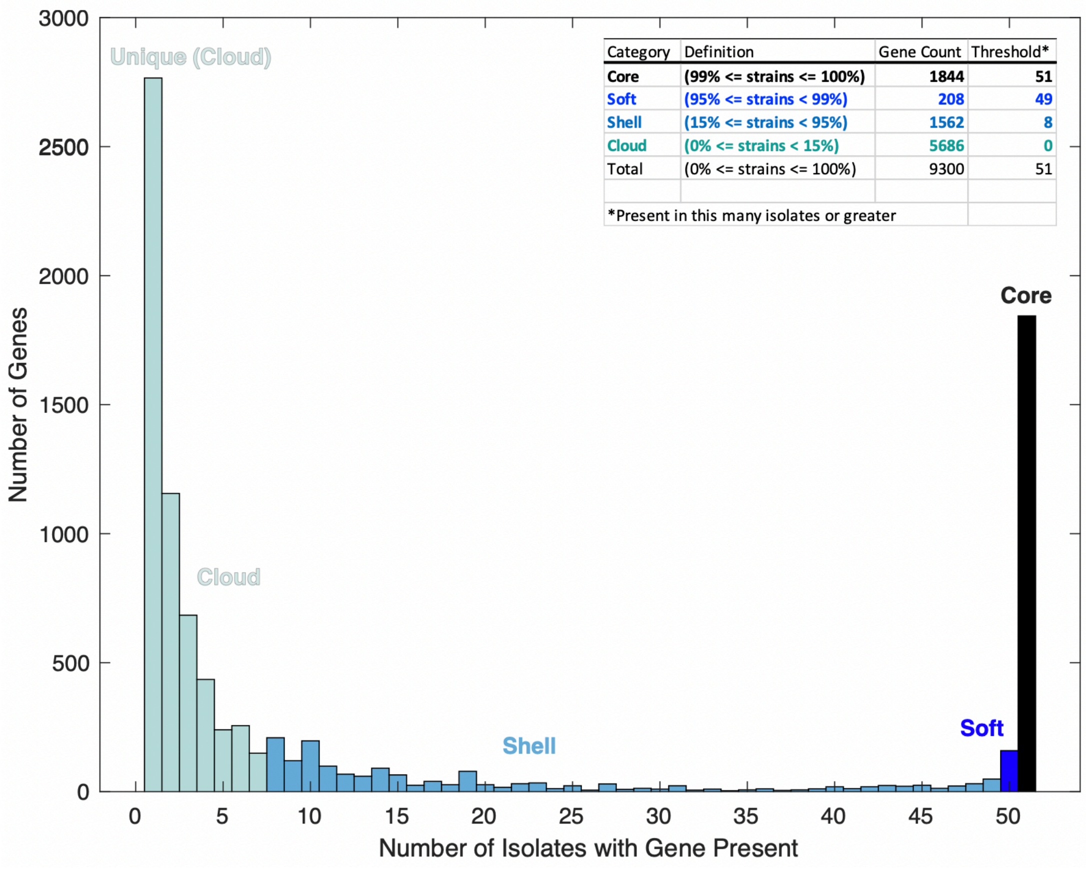

Figure S1. Gene membership of the 51 *Enterococcus faecalis* isolate genomes analyzed. Counts and categories as estimated by Roary analysis of presence/absence as described in the methods.

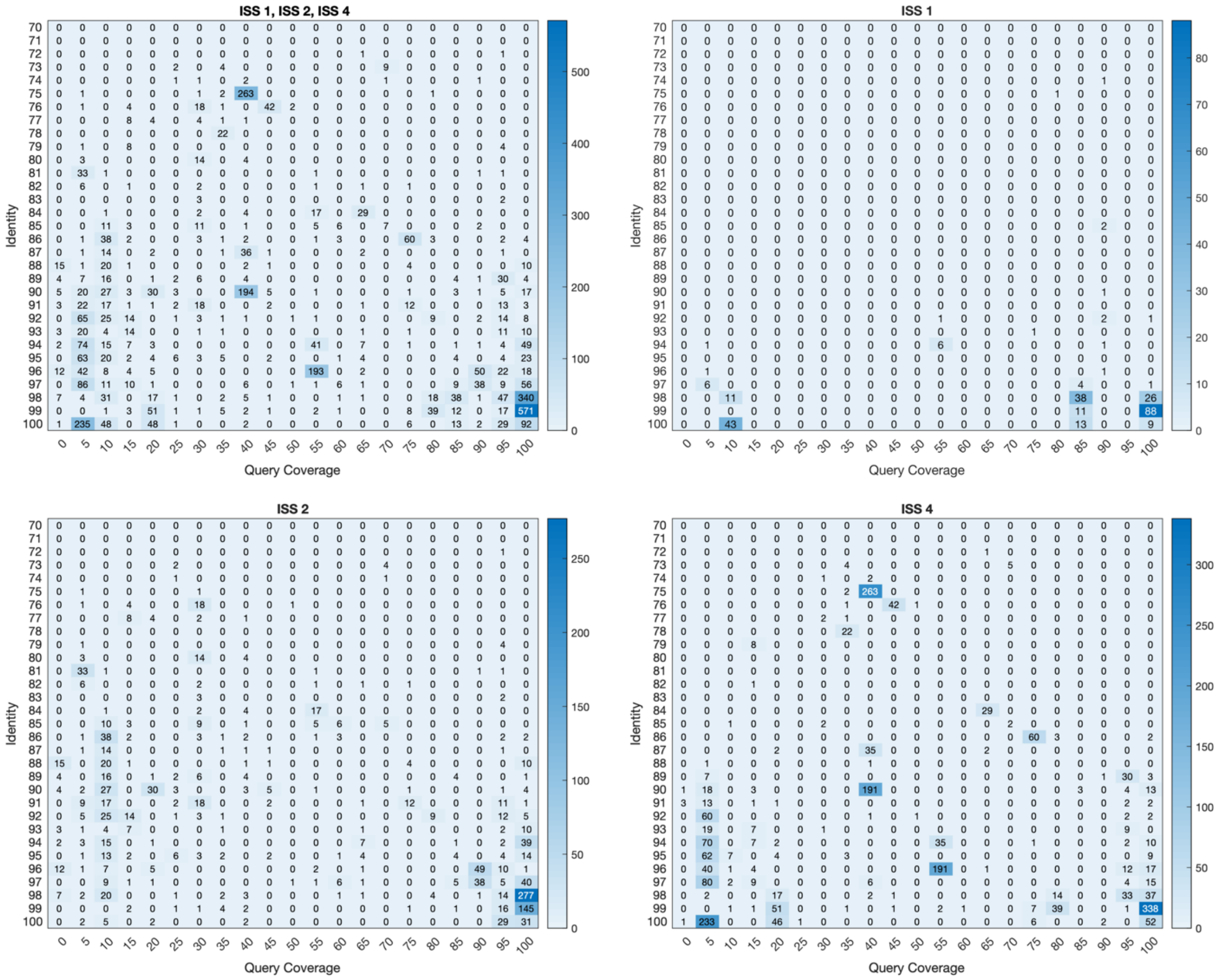

Figure S2. Sequence similarity of *Enterococcus faecalis* genes unique to ISS_1, ISS_2/ISS_3, and ISS_4. Query coverage and percentage sequence identity from BLASTn searches across all isolates (upper left) and for each isolate, respectively. Because ISS_2 and ISS_3 have no individually unique genes, and differ in their core gene alignment by only 14 bases, but have genes that are not represented in any of the other 49 genomes studied, ISS_3 was excluded from this analysis. Supplementary Data File 1 contains details of each BLASTn hit.

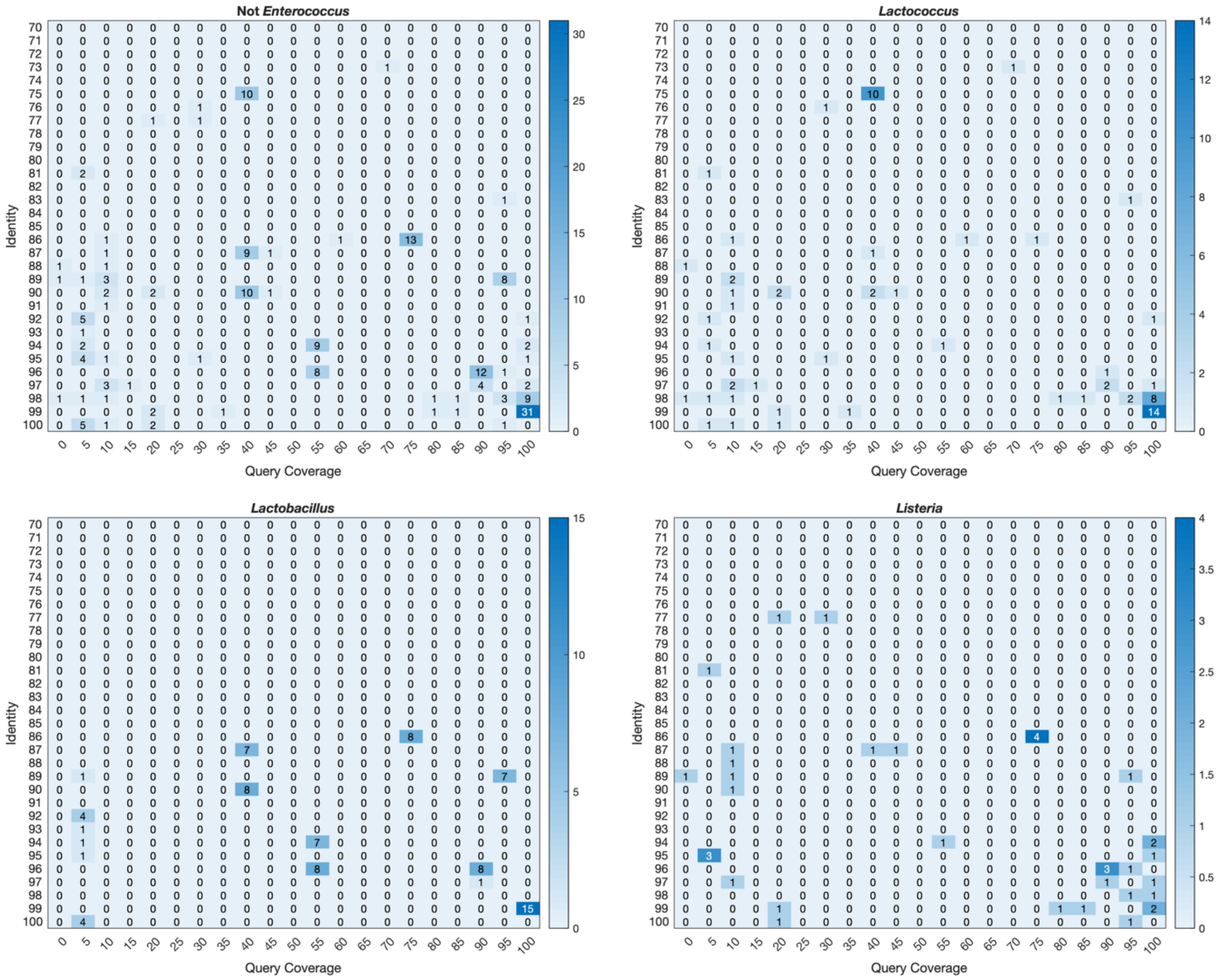

Figure S3. Sequence similarity of *Enterococcus faecalis* genes unique to ISS_1, ISS_2/ISS_3, and ISS_4 segmented by the genus of the hit subject. Query coverage and percentage sequence identity from BLASTn searches are as in Fig. S2 (upper left) after eliminating all hits associated with *Enterococcus* (upper left) and further segmenting by genus (there were only three remaining genera).

Table S1. Illumina HiSeq short read coverage estimates.

Strain	Raw Paired End Reads (x 10^6)	Raw Bases (x 10^8)	Estimated Coverage*
ISS_1	2.29	11.4	381
ISS_2	1.33	6.66	222
ISS_3	2.95	15.3	509
ISS_4	5.25	26.3	875

*Estimate genome size 3.0 Mb.

Table S2. Nanopore long read coverage estimates.

Strain	Median Read Length (kb)	Total Reads (x 10^4)	Read Length (N50) (x 10^3)	Total Bases (Mb)	Estimated Coverage*
ISS_1	1.76	3.48	5.41	106	35.2
ISS_2 R1, R2	1.84<br>4.25	5.45<br>1.22	6.53<br>11.6	188<br>81.3	62.7<br>27.1<br>89.8 total
ISS_3 R1, R2	1.76<br>5.24	6.17<br>1.83	6.94<br>11.6	214<br>133	71.2<br>44.4<br>116 total
ISS_4	1.97	4.51	6.93	165	55.1

*Estimated genome size 3.0 Mb. R1 = Run 1; R2 = Run 2;

Table S3. Predicted intact phage content as determined by PHASTER for ISS and control *E. faecalis* isolates (Arndt et al. 2016).

Isolate*	Length (Kb)	Score	Keyword	Total Proteins	Phage Proteins	Hypos	ATT Site	Most Common Phage Name (gene hit count)	Most Common Phage (% Identity)	GC Content (%)
ISS_2	41.1	130	integrase, recombinase, terminase, portal, head, coat, tail, lysin	66	52	14	yes	PHAGE_Entero_phiFL3A _NC_013648 (32)	48.5	34.2
	20.0	100	lysin, tail, head, capsid, portal, terminase	24	20	4	no	PHAGE_Lactob_Lj928. _NC_005354 (6)	25.0	36.9
	49.9	125	integrase, terminase, portal, head, capsid, tail	48	47	1	yes	PHAGE_Entero_phiFL4A _NC_013644 (46)	95.8	38.1
ISS_3	49.9	125	tail, capsid, head, portal, terminase, integrase	48	47	1	yes	PHAGE_Entero_phiFL4A _NC_013644 (46)	95.8	38.1
	20.0	100	terminase, portal, capsid, head, tail, lysin	24	20	4	no	PHAGE_Lactob_Lj928. _NC_005354 (6)	25.0	36.9
	48.2	130	lysin, tail, coat, head, portal, terminase, recombinase, integrase	65	52	13	yes	PHAGE_Entero_phiFL3A _NC_013648 (32)	49.2	34.6
ISS_4	21.7	110	terminase, portal, capsid, head, tail, transposase, lysin	25	21	4	no	PHAGE_Lactob_Lj928 _NC_005354 (6)	24.0	37.1
MMH 594	35.7	130	lysin, tail, capsid, head, portal, terminase, integrase	54	51	3	yes	PHAGE_Entero_phiFL4A _NC_013644 (48)	88.9	37.6
V583	21.7	110	terminase, portal, capsid, head, tail, transposase, lysin	25	21	4	no	PHAGE_Lactob_Lj928 _NC_005354 (6)	24.0	37.1

*No intact phages were detected in the genomes of ISS_1 or OG1RF.

Table S4. CRISPR-Cas system detection in the genomes of ISS and reference strains (Couvin et al. 2018).

Isolate	CRISPR Evidence Level	Start	Stop	Direct Repeat Length	Spacer Count	Cas Cluster Type
ISS_1	4	1459041	1460000	36	14	II-C
ISS_2	4	1608874	1609305	36	6	N.D.
ISS_3	4	1327888	1328319	36	6	N.D.
ISS_4	3	1234036	1234336	37	4	N.D.
	4	2514314	2515141	36	12	II-A
OG1RF	4	422486	422984	37	7	II-A
	4	1738187	1738685	37	7	N.D.

N.D.: not detected. No CRISPR loci were detected in MMH594 or V583.
